# Racial microaggressions and interculturality in remote Central Australian Aboriginal healthcare

**DOI:** 10.1186/s12939-023-01897-4

**Published:** 2023-05-25

**Authors:** Mary Wicks, Christine Hampshire, Jeannie Campbell, Louise Maple-Brown, Renae Kirkham

**Affiliations:** 1 Menzies School of Health Research, Charles Darwin University, PO Box 1294, Alice Springs, NT 0871 Australia; 2 Pintupi Homelands Health Service, PMB 145 Kintore via Alice Springs, Alice Springs, NT 0872 Australia; 3 Central Australian Health Service, PO Box 721, Alice Springs, NT 0871 Australia; 4 Menzies School of Health Research, Charles Darwin University, PO Box 41096, Casuarina, Darwin, NT 0811 Australia; 5 Department of Endocrinology, Royal Darwin and Palmerston Hospitals, PO Box 41326, Casuarina, Darwin, NT 0811 Australia

**Keywords:** Health Communication, Microaggressions, Interculturality, Discourse Analysis, Aboriginal Australians, Central Australia, Remote Health, Culture

## Abstract

**Background:**

An epidemic of type 2 diabetes in remote Aboriginal people in Central Australia, contributes to high rates of morbidity and mortality. Remote non-Aboriginal Health Care Workers (HCW) and the Aboriginal people they serve inhabit a complex cultural interface. This study aimed to recognise racial microaggressions in the everyday discourse of HCWs. It proposes a model of interculturality for remote HCWs that avoids racialisation and essentialising of Aboriginal people’s identities and cultures.

**Methods:**

Semi-structured in-depth interviews were undertaken with HCWs from two Primary Health Care services in very remote Central Australia. Fourteen interviews were analysed from seven Remote Area Nurse, five Remote Medical Practitioners and two Aboriginal Health Practitioners. Discourse analysis was employed to explore racial microaggressions and power relations. NVivo software assisted in the thematic organisation of microaggressions according to a predefined taxonomy.

**Results:**

Seven microaggression themes were identified - racial categorization and sameness, assumptions about intelligence and competence, false colour blindness, criminality and dangerousness, reverse racism and hostility, treatment as second-class citizens and pathologizing culture. A model of interculturality for remote HCWs was based on concepts of the third space, deCentred hybrid identities and small culture formation on-the-go combined with a duty-conscious ethic, cultural safety and humility.

**Conclusions:**

Racial microaggressions are common in the discourse of remote HCWs. The model of interculturality proposed could improve intercultural communication and relationships between HCWs and Aboriginal people. This improved engagement is required to address the current diabetes epidemic in Central Australia.

**Supplementary Information:**

The online version contains supplementary material available at 10.1186/s12939-023-01897-4.

## Background

The ‘dusty encounter’ that eminent Australian anthropologist WEH Stanner wrote about in 1958 continues to be an apt way to describe the continuing miscommunication between non-Aboriginal Health Care Workers (HCW) and Aboriginal people living in remote communities of Central Australia. It has been suggested that this unknowing continues and in very remote Aboriginal Australia, where Aboriginal and western logic meet most starkly, a ‘mutual incomprehension’ [3] results. Yet as philosopher Hannah Arendt alludes to in her iconic work, The Human Condition, [2] both difference and sameness are required for the possibility of dialogue and a real knowing of the Other. Anthropologist Melinda Hinkson refers to this quandary as ‘difference-yet-relatedness’, with difference marking life-ways of remote Aboriginal Peoples despite an increase in relatedness with the wider Australian and globalised society [4]. This reflects the intercultural move in the anthropology of Indigenous Peoples away from the conceptual interface of distinct inviolable cultures and toward an understanding of the mutual interpenetration of Indigenous and non-Indigenous sociality [4, 5].

### Identity

Formulations of identity, both individual and group, are important in the consideration of the intercultural. Identity is contextual, constructed and provisional [6]. ‘Identity fundamentalism’ [7] describes individuals or groups who claim, or are attributed, a single identity, for example their ethnic culture or religion, as their sole defining attribute. At the least this encourages stereotyping. Cultures and identities that are ‘airtight, impermeable, homogenous,’ can veer into ethnocentrism, totalitarianism, and nationalism [8]. This fundamentalist identity has been described as the ‘delusion of singular identity’ [9] and ‘the danger of the single story’ [10] referring to the reductionism inherent in conceptualising people as having a sole identity.

Aboriginal identity in the (post)colonial setting of Australia is complex. Historically the identity of Aboriginal peoples, where it survived policies and practices of violent and passive eradication, has been denigrated or suppressed [7]. Noel Pearson, an Aboriginal intellectual from Cape York in Queensland, argues for the concepts of ‘layers of identity’ and ‘communities of identification’ [11]. He urges Indigenous people, especially the young, to embrace their multiple identities. Pearson points to the additive nature of ethnic identity, where ‘one’s own British or European or Asian or African ethnic identification does not diminish one’s identification as Indigenous’ [7].

This layered identity espoused by Pearson is not universally accepted [12, 13]. Re/claiming a strong Aboriginal identity can help build personal resilience and a sense of belonging, improving mental health and well-being for Aboriginal people [14, 15]. It is also argued that a ‘demonstrably Indigenous’ [16] personal and cultural identity needs to be projected to further the politics of pan-Indigenous solidarity, and as a statement of continuing Indigenous survival [13]. This is pertinent in contemporary Australia as it negotiates long suppressed issues of national Indigenous recognition [17].

### Interculturality

The history of humanity is one of interaction across cultures, languages and geographical borders [18]. Rather than just a set of skills and knowledge, interculturality is better understood as a stance; a qualitatively different way of being-in-the-world [19, 20]. This requires an appreciation of our natural hybridity as modern beings as we inhabit multiple identities and live in hybrid complexity [18, 19]. The dichotomised identities of indigeneity, the ‘we’ and the ‘us’, vis-a-vis ‘them’, are false as there is no ‘us’ or ‘them’ only real embodied lives lived dynamically in particular places and times [6]. Interculturality is, amongst other things, a ‘finding ourselves in others and others in ourselves’ [20], even in the remote Australian context where cultural difference can be experienced as a radical alterity [6, 21].

### Racism

Pearson describes ‘the original sin’ of racism against Aboriginal people since early colonisation with assumptions of innate inferiority and sub-human status. Sadly, he says, ‘we are a much unloved people. We are perhaps the ethnic group Australians feel least connected to’ [7]. Racism presents a major challenge to both Aboriginal health and interculturality. Literature on racism and Australian Indigenous health has broadly replicated international findings of poorer health outcomes for those who experience racism [22–26]. In a recent national cross-sectional study, 58% of Indigenous people reported having experienced racial discrimination in the previous 12 months [23]. Racial discrimination was associated with an increased prevalence of diabetes, hypertension, dyslipidaemia and heart disease. This effect appeared to be dose-related with higher prevalence of dysmetabolic indicators in those with higher racial discrimination scores [23]. A sub study in Central Australia found 71% of Aboriginal people reported experiences of racial discrimination and this was found to be highest in the regional town of Alice Springs as compared to remote communities [24]. Despite this data, many Australians reject the notion that they are racist, equating it to the extreme expression seen in white supremacists, and rejecting the idea that ‘good people’ can be racist [27].

Whilst race is no longer coherent as a biological or genetic reality, it can be understood as socially constructed, [28] which does not make its effects less real [29]. As a concept, race carries a dark history from marginalisation to genocide. As a societal system it expresses, both explicitly and implicitly, beliefs and attitudes that perpetuate inequalities through maintaining differences in power, resources and opportunities [30]. Communication issues continue to loom large in remote Central Australian healthcare with a ‘pervasive failure to communicate,’ [31] that threatens cultural safety, [32] and impedes access to healthcare, [33] continuing in contemporary health settings. Discourse is crucial in the reproduction of interpersonal racism [28]. This ongoing complaint of poor communication by HCWs does not necessarily imply malicious intent, rather it points to racism as extant in the health system [34] and other institutions in contemporary Australia [35].

Overt racism may not be as prominent as in the past, but covert racism, or ‘everyday racism’, being more subtle and pervasive, continues unabated [36]. Racial microaggressions are another way to conceptualise everyday racism directed toward racialized individuals or groups. They ‘convey the everyday verbal [or] nonverbal…slights, snubs, or insults, whether intentional or unintentional, that communicate hostile, derogatory, or negative messages’ [37]. Everyday racism ‘can be perpetrated perfectly well, with a pleasant smile, and with good manners’ [35]. The ‘micro’ prefix and unconscious intent do not ameliorate the impact of microaggressions on racialized person/s. The therapeutic relationship is vulnerable to racial bias, which can lead to the ‘paradox of well-intentioned [clinicians] providing inequitable care’ [38]. This opaque view of the HCW in perpetuating everyday racism needs confronting to ameliorate the burden of racism on Indigenous people and to call attention to the power differential inherent in ‘white privilege’ [39, 40].

There is a paucity of research into how racial microaggressions are enacted by Australian HCWs who work with Indigenous people. Shaburdin and colleagues report a study of racial microaggressions in the discourse of health workers serving Aboriginal, refugee and immigrant groups in rural Australia [30]. They found racial microaggressions common in HCW discourse, and identified three of the most common: problematising of culture, implied cultural superiority of HCWs and relegation of responsibility by HCWs as a self-justified response to ‘difficult clients.’ They concluded that the values and attitudes of the dominant western culture were expressed through the health system and reinforced through racialized language, and systems of management and administration.

Microaggressions are not only racial. They can be targeted toward any person in a marginalised group, for example in the areas of gender, sexual orientation, disability, class and religion [37]. Intersectionality theorises that inequality can be compounded by multiple systems of inequality (e.g., Aboriginal and woman) and that these people can experience worse outcomes than if they had a sole axis of disadvantage [41]. Due to a certain siloing of marginality, the needs of these individuals may not be adequately addressed (for example by anti-racist or feminist discourse alone) [42].

### Cultural safety and humility

In a recent review of international definitions of cultural safety and cultural competency Curtis and colleagues sought to identify the most effective model in the cultural training space. They recommended a move to cultural safety with its foregrounding of power differentials both at the interpersonal and systemic levels [38]. An abbreviated definition from the First Nations Health Authority in British Columbia states that ‘cultural safety is an outcome based on respectful engagement that recognizes and strives to address power imbalances inherent in the health care system. It results in an environment free of racism and discrimination, where people feel safe when receiving health care’ [43]. They combine cultural safety with cultural humility where cultural humility ‘is a process of self-reflection to understand personal and systemic conditioned biases, and to develop and maintain respectful processes and relationships based on mutual trust…[it] involves humbly acknowledging oneself as a life-long learner when it comes to understanding another’s experience’ [43]. The combination of these two concepts of cultural safety and cultural humility embodies what is required for a quality intercultural presence. This dual focus has been advanced by the Congress of Aboriginal and Torres Strait Islander Nurses and Midwives forming the basis of their intercultural learning programme directed at non-Indigenous nurses and midwives [44].

### Study context

This study was undertaken with HCWs in two remote Aboriginal health clinics in Central Australia. This region covers an area of more than half a million square kilometres with a small, dispersed population of 41,000 people. The two remote Aboriginal communities in this study were medium sized with populations of approximately 420 and 580, with more than 90% of people identifying as of Aboriginal descent [45]. Socioeconomic indices in very remote communities of the Northern Territory point to these Aboriginal people as among the most materially deprived people in Australia [46].

The primary healthcare clinic is one of several western institutions in remote Aboriginal communities that are invariably managed and staffed by non-Aboriginal people. Clinical staff include several Remote Area Nurses (RANs) and a visiting Remote Medical Practitioner (RMP). Some clinics have Aboriginal Health Practitioners (AHP) who are generally also residents of the community. These clinics provide primary healthcare as well as on-call acute care services 24 h a day.

## Rationale

An epidemic of type 2 diabetes in remote Aboriginal people of Central Australia, and its corollaries of cardiovascular and renal disease, is currently one of the biggest obstacles to Closing the Gap in health disparities between Aboriginal and Torres Strait Islander people and other Australians [47]. Sustained, high quality, therapeutic relationships between HCWs and Aboriginal people are required to facilitate shared decision making and support chronic condition management self-efficacy [48]. This requires relationships that are welcoming, respectful, and accepting [48] which in turn require quality intercultural presence and skills. Thus, diabetes prevalence and outcomes are, in part, related to experiences of racism and the amelioration of racism is tied to concepts of cultural identity and interculturality.

## Aims

- To explore racial microaggressions in the discourse of HCWs working with Aboriginal people in very remote health settings, and,
- To propose a model of interculturality for remote non-Aboriginal healthcare workers.

## Methods and positioning

Discourse is a form of meaning making within a specific social and political context. Analysis of discourse sheds light on the social construction of reality, knowledge, identity and power relations [49]. In this study discourse analysis was utilised as a means of identifying racialized discourse about ‘ethnically different others’ in the conversation of remote HCWs, which involved ‘negative other-presentation and positive self-presentation’ [28].

This qualitative study included semi-structured, in-depth interviews with remote HCWs from two Central Australian Aboriginal communities. The communities represent two Aboriginal language groups (Pintupi-Luritja and Anmatyerre), and health service types (government service and Aboriginal Community Controlled Health Service). Both communities are situated in very remote Central Australia (ARIA+) [50].

The interview guide (see Supplementary material) was informed by the following constructs: models of health, perception of HCW role, cultural understandings, communication issues and experiences of working with Aboriginal people. The interviews occurred during five visits between April 2021 and August 2022. All HCWs in the clinics were invited to an interview, assured of confidentiality, and provided informed written consent regarding the study aims and processes. All interviewees were proficient in English and agreed to audio recording of the interview. Two RMPs were interviewed together as were three RANs at their request.

The interviews were conducted and transcribed by one researcher (MW), who has worked both as a RAN and as a visiting specialist physician to remote Aboriginal clinics for > 15 years. Findings were cross-checked for interpretation of meaning with two other clinical researchers (CH & JC) who both work in the same context as the participants. CH is a RMP who has worked for 16 years in remote Aboriginal health. JC is a AHP of 22 years service who identifies as an Anmatyerre Aboriginal woman. She provided an additional perspective regarding interpretation of Aboriginal cultural and Aboriginal Health Practitioner content. All researchers identify as female.

## Data analysis

Coding analysis was performed utilising NVivo software [51] and initially progressed deductively according to the interview themes. A second coding proceeded inductively utilising the taxonomy of racial microaggression expounded by Williams and colleagues to categorise identified microaggressions thematically [52].

## Ethics approval

This research was carried out in accordance with Australia’s National Health and Medical Research Council’s ethical principles of the National Statement on Ethical Conduct in Human Research 2007/2018 [53]. Approval was gained by the Central Australian Human Ethics Research Committee: Reference number CA-20-3917. This research sits within the Diabetes Across the Lifecourse: Northern Australian Partnership which has an Aboriginal and Torres Strait Islander Advisory Group who provided project oversight.

## Findings

Fourteen HCWs were interviewed. Participant characteristics are outlined in Table 1.

**Table 1:** Characteristics of Participants

	AHP	RAN	RMP
**N**	2	7	5
**Age, average (range) years**	47.5 (45–49)	57 (29–66)	61 (39–72)
**Male Sex n (%)**	0	2 (28)	1 (20)
**Time remote average (range) years**	26	5.9 (0.6–11)	13.8 (7–20)
**Time current role average (range) years**	18	2.6 (0.08–7)	7.6 (4–10)
**Permanent Staff**	2	6	5

AHP: Aboriginal Health Practitioner; RAN: Remote Area Nurse; RMP: Remote Medical Practitioner

The racial microaggressions identified in the talk of HCWs were grouped into seven categories according to the definitions proposed by Williams and colleagues [52]. The exemplar talk and the implied subtext are discussed, with additional extracts displayed in Table 2.

### Racial categorization and sameness

Racial sameness, or an essentialist model of culture, was expressed through RAN2s statement; *you know what they’re like!* This stereotyping uses collusion to suggest that all Aboriginal people are the same in their behaviours and choices. AHP1 felt that AHPs were denied the opportunity to represent themselves to new RAN staff. New staff had already been briefed by RANs regarding the identity or personality of the AHPs prior to meeting them and *without letting them find out what we’re like.*

### Assumptions about intelligence, competence or status

Statements based on assumptions of lower intelligence or incompetence were also based on racial stereotypes. RAN3 commented that AHPs were *directed that [they] have to just go and drive the car* instead of being utilised as providers of clinical care, for which they had been trained. She then notes that AHPs are not *encouraged to work to their full potential … so they don’t.* In a more general remark about Aboriginal people RAN1 questioned; *do they [Aboriginal people] know what they want? I think here that the people don’t really know what they want.* This statement suggests that Aboriginal people lack agency over their desires and actions. RMP3 recalls her experience with non-Aboriginal HCWs from poor migrant backgrounds who *expressed horror that Aboriginal people aren’t making better of their circumstances.* This suggests that Aboriginal people lack a certain intelligent motivation or capacity to make improvements to their life if desired.

### False colour blindness/ invalidating ethnic identity

HCWs *not seeing colour* and treating *everybody the same* (RAN4), may sound egalitarian, however, the attitude of equality, treating *everybody the same* obfuscates the notion of equity where people are, rather, given according to their need. Not seeing colour can also invalidate the issues that a person of colour may experience because of their very colour, such as racial discrimination. When discussing the suffering that local Aboriginal people had undergone since colonisation RAN4 agreed but added that *there’s also lots of sad things with white fellas*. This truism can function to invalidate and normalise the intergenerational suffering of Aboriginal people which was the issue under discussion at the time.

### Criminality or dangerousness

Aboriginal people were sometimes cast as dangerous people and new RANs were advised by those orientating them to avoid relationships with Aboriginal people; *‘don’t be friends’ … ‘you’re only there to provide a service.’* (RAN3) In one community Aboriginal people requesting care afterhours would present themselves to the house of the on-call RAN. This had recently changed due to COVID-19 distancing procedures and after-hours calls were being triaged by telephone. RAN5 expressed that she *was really scared…I felt quite scared*, with the practice of having those in need present to their residence. RAN8 also expressed this fear ‘… *because [they’re] coming to your house. It was all hours. It was scary*.’ Whilst this procedure is no longer considered best practice, the subtext of this conversation was that the fear induced by people presenting to an on-call HCWs personal residence was greater for Aboriginal than non-Aboriginal people.

### Reverse racism / hostility

Reverse racism and a certain hostility were detectable in the talk of RANs. Perceptions that Aboriginal people receive special treatment because of their ethnicity were common.

*RAN1: I’ve been to lots of communities, and I would say that [community x] is the most demanding …that expectation…*

*Interviewer: that you will look after?*

*RAN5: Yep…*

*RAN1: They’re that spoiled …I think…*

*RAN5: And they’ve been treated well…*

*RAN1: And they’ve been spoiled here…*

*RAN5: I think they’re spoilt…*

The impression that Aboriginal people were spoilt decries the reality of the decimation of colonial occupation to people’s lifeways and the ongoing poverty and marginalisation that continue. The lack of deference of Aboriginal people to white RANs caused indignation; RAN5 says ‘… *like I’m here to try and empower you and now you’re telling me it’s my job!’*

RMP1 told of RAN complaints about an AHP:

*The nurses complained about her [AHP] and said that she wasn’t working. Whereas I thought she worked really hard. From my perspective, she would do observations, and she spent most of the day sitting out on the veranda talking to the older people and talking to people. And so, people will come into the clinic as a safe place and sort of a meeting place. And from my point of view, that’s the job I didn’t ask her to do, but that’s the job I wanted her to do!*

In this instance the RANs perceived the AHP as lazy and not pulling her weight as a team member. In contrast the RMP understood the contribution of the AHP as valuable and unique. RMP5 recounted *resentment [expressed by a RAN] that ‘you’re [AHP] getting paid, and you’re not working as hard as me or you’re not giving up as much as me, and I’m tired and burnt out anyway’. So, you know, ‘they never come, they never blah, blah, blah’.* In these cases, AHPs are accused of being lazy and not having the same level of commitment to their role as the RANs.

### Pathologizing culture

Attribution of poor health, suboptimal health behaviours and decisions to a pathologized Aboriginal culture was a common topic of talk. Culture was also implicated as giving licence to what was interpreted as dependent behaviour of Aboriginal people, expecting RANs to be *at their beck and call*:

*RAN3: I think they’re a lot more dependent in [community x]. Some people kind of expect us to be at their beck and call.*

*Interviewer: do you think that’s a cultural thing or a personal thing?*

*RAN3: No, I think it’s cultural.*

For RAN1 Aboriginal people did not care about their health:

… *in the same way ... the same structured way that we do ... but because they’re so nomadic they just go from one place to another, and they just lose that momentum. They haven’t got that systematic structure. I don’t think it’s not that they don’t want to.*

For RMP2 … *[culture] hinders [health] for a large number [of people].* This belief that cultures and behaviours attributed to culture, work actively against good health was also expressed by RMP4 who signified a difference between traditional culture and current cultural circumstances. *Well, you’ve got traditional culture - probably doesn’t hinder health. Your amalgamation of cultures in the current time probably does.*

### Second-class citizens

Some AHPs were aware of being treated as second-class citizens and lamented its occurrence; AHPs were *unappreciated and unsupported; I don’t think they felt very valued* (RMP3) and not *looked at as part of the team* (RAN2). AHP1 felt HCWs *talk[ed] down to … not treating us … as professionals;* and being treated as HCW property, *they say, ‘my health workers…my this, my that …’* Non-Aboriginal HCWs noted this treatment of Aboriginal workers and expressed that *it was a real pity, and I haven’t seen [the Aboriginal workers] since* (RMP3*).*

### Unequal power relationships

Whilst not a microaggression, unequal power relations were often markers of underlying racial discrimination. Some RAN talk acted to assert dominance over, and ownership of, clinic resources. For example, Aboriginal people travelling away from the community without taking a supply of medications was couched in the possessive pronoun ‘my’; *They go without prioritizing that ‘my’ medications are important* (RAN6). Most of this talk centred around the use of clinic motor vehicles to assist in the transport of Aboriginal people to the clinic. In one of the study communities this was a significant issue with the distances people had to travel between 10 and 100 kilometres in a region with no public transport options. Again, the personal pronoun ‘I’ exerts control when RAN6 chose to send a vehicle to pick up the Aboriginal person *that I need … to come in …* rather than what the Aboriginal person might see as a priority. This issue of transport was often couched in the frames of irresponsibility and dependence of Aboriginal people; *it just becomes a learned behaviour if you like - they want to get picked up* (RAN3), with little or no reflection on the material poverty in which most Aboriginal people lived. As RMP3 reflected with some distress, *these people are living in abject poverty … this is a big ask just expecting people are fully fuelled up, but, ‘well, they’ve got enough fuel to get to the Roadhouse.’ It’s just awful!*

**Table 2:** Racial Microaggressions based on taxonomy of Williams and colleagues [52]

Microaggression Theme	Discourse Example	Subtext Message
**Racial Categorization and Sameness**	RAN2: You know what they’re like…	Collusion regarding Aboriginal people - ‘They are all the same.’ Stereotyping.
**Assumptions about Intelligence, Competence or Status**	RAN4: Yeah, because it’s us telling them [AHPs] what to do all the time … I haven’t had any issues with the girls.	AHPs are not equal peers. Infantilising AHPs with language of ‘girls’.
**False Colour Blindness/ Invalidating Ethnic Identity**	RAN4: I always treat everyone exactly the same; doesn’t matter what colour.	Implies equality. Equality is giving each one the same whilst equity is giving each one what they need.
**Criminality or Dangerousness**	RAN3: And well I’ve been particularly told, ‘Don’t ever think that’…like, ‘don’t be friends’, that ‘you’re only there to provide a service.’	Friendships with Aboriginal people are inappropriate and can be dangerous.
**Reverse Racism / Hostility**	RAN1: Personally, I think that the white fella came in and started taking care of the people. And now that’s the expectation that we are, in some ways, subservient to be honest. Because a lot of clients will come to the room [and] sit down. They’re quite capable [of getting] a cup. ‘Give me a cup, a cup of *Kapi’* (water). I think [they’re] definitely, they are on a higher level than us. To be honest. We are here to, in some ways, in some people’s minds, to serve.	HCW annoyed that she is seen as one who serves despite her professional ranking and whiteness.
**Pathologizing Minority Culture or Appearance**	RAN1: … what’s the use of growling at them? It’s hopeless. It’s their choice!	Aboriginal patients make the wrong health choices due to their ethnicity and this is not amenable to *growling* (a euphemism for chastising).
**Second-Class Citizens /** **Ignored & Invisible**	RMP1: She [AHP] did leave, and she felt unappreciated and unsupported, of course, because … she was constantly an outlier and never got anything.	Aboriginal people excluded or treated as less valued health professionals than other team members.
* Unequal Power Relationships	RAN6: Most times it’s because they’re transient. They go without prioritizing that **my** medications are important.	The clinic has the resources, and the RAN is the gatekeeper.

* Not a specific category of racial microaggression

## Discussion

The participants in this study varied in regard to time in remote practice and time in current role. For RANs the average time of both was higher than reported in focussed studies of RAN retention in this region [54]. The difference was driven by the retention of RANs within the Aboriginal Community Controlled health service (6.5 vs. 3.8 years). Due to the small sample size, it is not possible to predict what effect this may have had on the talk of RANs reported in this study. Only one of the 14 HCWs was on a short-term agency contract which is again unusual for this region in which there is a high deployment of this type of short-term worker [54]. Given only two health services participated in this study, no conclusions are possible regarding the effects on HCWs working in the different health service types.

Remote non-Aboriginal HCW talk included a significant burden of racial microaggressions. In this study it was most notable in the discourse of RANs. RAN talk adhered to van Dijk’s sociocognitive model of prejudice where,

*Prejudice is both a cognitive and a social phenomenon. It is … a shared form of social representation in group members, acquired during processes of socialization and transformed and enacted in social communication and interaction. Such ethnic attitudes have social functions, e.g. to protect the interests of the ingroup* [55].

van Dijk recognises two forms of racist discourse; that directed at ethnically different Others and that about ethnically different Others [56]. This study focussed on the second type of racist discourse as expressed in racial microaggressions, and found within this discourse resonances of a shared prejudicial social representation of Aboriginal people. This raises questions about the socialisation of remote HCWs and the challenges to leadership in helping to facilitate more liberating and less prejudicial conceptions of Aboriginal people.

While there is widespread agreement that race and ethnicity are socially constructed, the role, if any, of the ‘idea of race’ [29] is unclear. Noel Pearson argues for the concept of one race, that of humanity, to which we all belong, [7] which is biologically correct but belies both the historical and contemporary functions of race. Race or ethnicity continues to be used as a proxy for many measurable characteristics and the ‘biology of race’ remains extant in the health field. In Central Australia for example, Aboriginal descent is considered a risk factor for diabetes, renal and heart disease. This unthinking operationalisation of ethnicity/race as a risk factor presumes an internal biological predisposition to, usually, poorer outcomes [57]. It is racism, rather than race or ethnicity, that has garnered the scientific evidence of contributing to biological harm [58]. Ethnicity is more accurately conceived as a ‘risk marker of vulnerability, bias or systematic disadvantage’ [57].

Skin colour, as the ‘optics of race,’ [57] has been central to determining ingrained ideas of racial difference. A claim of ‘colour-blindness’ by HCWs suggests that racial labelling, with its biases, has been transcended and the HCW has emerged on a higher plane of post-racialism where the individual before them is seen for who they are, their character and qualities [59]. Unfortunately this approach can impede racial equality rather than foster it [35] and can make the HCW less sensitive to recognising racial discrimination. Asserting colour-blindness dismisses the lived experience of a person of colour, and how that has been shaped by racism, both socially and biologically. It is also dismissive of the role of racism in health [59]. With Aboriginal people in this region having the lowest socioeconomic status in Australia [46] claims of equity have even more purchase. Instead of promoting colour-blindness as an answer to racial equality, a ‘race-conscious medicine’ allows HCWs to address structural racism and embody a clinical praxis that aims to reduce racial disparities and the barriers that cause them [29].

The intercultural stance proposed by Aboriginal intellectual Pearson offers a theoretical basis in which respectful interculturality can be formulated. This model requires a questioning of received ideas about culture and the Other and allow for a re-imagining of the Self-Other relation. In his critique of the reductionist popular contemporary view of identity, Pearson proposes the concept of ‘layered identities’ or ‘communities of identification’ as workable metaphors of how peoples with varied identities can come together [11]. He is primarily concerned that Aboriginal people be able to enjoy the fullness of the myriad of identities available to them in any given context, and that the ‘young are not told what is Indigenous and what is not’, and are not cowered by prescriptive identity politics [17]. At times these identity layers may be in conflict with each other but this should not lead to essentialism or reductionism but rather to reason and dialogue. Borrowing from political scientist Robert Putnam’s social capital theory, Pearson describes strengthening relationships within relatively homogenous groups as ‘bonding’ ties and between heterogenous groups are ‘bridging’ ties:

*Bonding ties are important because they give expression to primary and proximate relationships in society. Bridging ties are important because they build and they increase recognition of wider affiliations between individuals right across society – even between cultural strangers* [11].

In the context of remote Aboriginal health clinics, a fundamentalist view of Aboriginal culture or identity causes a weakening of the bridges of identification that connect us to each other [7]. It risks an ‘Indigenous Australian identity [that is] so intimately connected with the organisation of … traditional society, that it will cease to exist if [they] embrace modernity’ [11]. The concept of bridging ties may help to address the silo-effect of marginalisation and better address the intersectionality needs of Others [42].

Interculturalist Adrian Holliday refers to the identity fundamentalism of cultures as ‘essentialist blocks’. These are imposed structures that ‘confine and reduce people … to prescribed stereotypes’ [20]. He advocates a deCentring methodology and praxis as a ‘crucial positioning’ of interculturality, as opposed to the ‘dominant, positivist, essentialist, racist large-culture thinking’ [19]. This deCentring implies a third space which is one of identity hybridity. It is not a totalising unity or middle/common ground, but a place where new relations of the Self and Other can develop and where an ‘ambivalent and hybrid process of meaning negotiation’ can occur [60]. Within this hybrid space one discovers ‘threads’ which are ‘resonances between ourselves and others’ that pull in opposition to essentialist large-culture blocks [19]. Threads point to the quality that allows genuine intercultural engagement. They are found in recognising our naturally hybrid identity and past experiences of interculturality that can be re-membered to help resonate with Others [20]. Holliday proposes ‘small culture formation on-the-go’ as the work of the deCentred third space [19, 20]. These are fleeting, transient cultures that can be constructed anywhere and are the spaces in which interculturality comes about. They occur when people come together intentionally to engage with each other. Constructing culture ‘on-the-go’ implies that we and everything about what we encounter is on the move with shifting multiple connections.

### A praxis model of interculturality

**Fig. 1:**
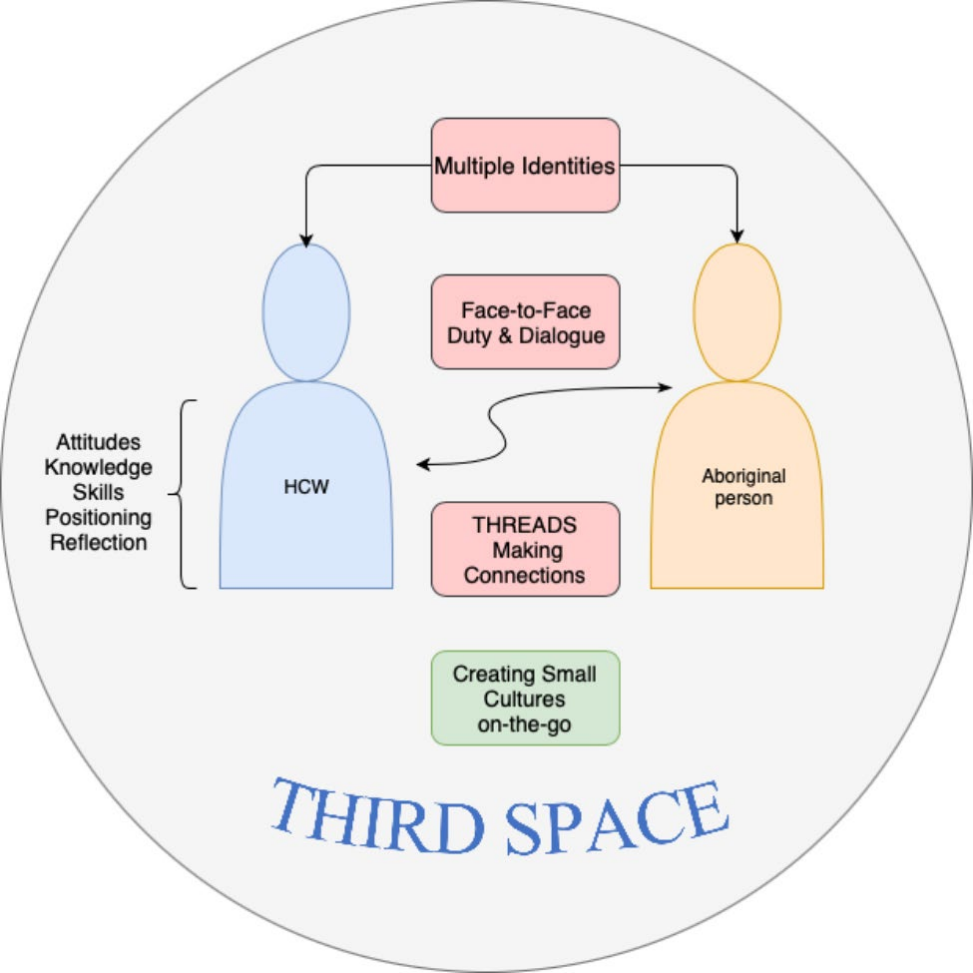
Intercultural praxis model for Central Australia

A model of intercultural praxis for remote Aboriginal healthcare settings is proposed (Fig. 1) using the major elements of Holliday’s deCentring methodology [19] and the ethical stance of the face-to-face espoused by philosopher Emmanuel Levinas [61].

#### Attitudes

The deCentred hybrid self requires specific attitudes when embarking upon the intercultural quest. A sense of curiosity and a desire to know the Other leads one to show interest in the intercultural encounter. A basic openness to the process, which may require changes in one’s self-understanding, requires a growth rather than a fixed mindset [62]. Adopting a cultural humility focus as defined earlier allows for an interpersonal stance that is Other-oriented rather than self-focused. It emphasises the skills and attitudes of openness, humility, self-reflection and life-long learning. Together with cultural safety, cultural humility requires a foregrounding of power imbalances inherent in the therapeutic relationship and a questioning of the assumptions of one’s own sociocultural identity. Assumptions held about Aboriginal people, and how these beliefs and behaviours are implicated in continuing Aboriginal disadvantage, can be exposed [63]. Importantly nurturing these attitudes and insights is a lifelong process not a discrete endpoint of cultural expertise [64].

#### Knowledges

To avoid cultural essentialism, it is important to do so from a place of basic knowledge. The intercultural process requires some understanding of Aboriginal history and cultures in Central Australia. Ignorance cannot exist regarding the: colonising history and its intergenerational legacy of poverty and trauma; basic cultural mores and protocols; the impact of racism and misconceptions regarding affirmative action policies. Indeed, ignorance about one’s own ignorance [63]. Capacity-building measures directed at non-Aboriginal HCWs are needed to address these basic knowledge gaps and to invert the dominant discourse of White people as the experts and builders of Aboriginal capacity [63].

#### Skills

The language spoken during an intercultural encounter speaks to power. There are 13 distinct, commonly spoken, Aboriginal languages in Central Australia, yet health discourse is dominated by English. The HCW is encouraged to learn some basic words and language structures of the people she is working with as a sign of openness and a relinquishment of power. It is recognised that a HCW is unlikely to become fluent in an Aboriginal language, so it is important to learn how to use Plain English in communicating with Aboriginal people whose first language is not English. There are ample local resources available to assist with the development of these skills [65]. Health services could facilitate the improvement of communication and Aboriginal language skills of their HCW employees by offering paid leave to attend and incorporating inter-cultural communication into compulsory assessable continuing education components. The development and practice of reflexivity is a core skill in interculturality. By ‘making aspects of the self strange’, one critically appraises one’s actions, thoughts, beliefs, values, cultural identity and power with a view to the effect these have on others, situations, and social structures [66].

#### Positioning

Positionality is a relational contextual concept. It is fundamentally about the intersection of social categories and power. From where do we speak, understand, act, and make sense of our world? Who is silenced, whose language is privileged and whose actions are dismissed or marginalised? ‘One’s position within social relations of power produces different standpoints from which to view, experience, act and construct knowledge about the world’ [67]. This positioning must be owned and kept front of mind during intercultural encounters.

#### Third space

With the above attitudes, knowledges, skills and reflection on one’s own positionality the deCentred HCW consciously chooses to inhabit a third space where the challenge is to think and do differently [19] or *otherwise* [68] from western cultural assumptions. This space is a symbolic in-between space that offers generative potential beyond dualistic conceptions [60]. It may not be easy to find a third space within the didactic of health communication-as-usual, and it can be an uncomfortable place to be, yet it is worth striving towards. In this space, ethnically or otherwise diverse and different Others are appreciated as hybrid identities and not constrained by essentialised presumptions of culture or other identifications. The HCW tries to find threads or resonances that are shared but does not try to control the meanings these threads may have to Others.

#### Face-to-face ethics

The relationship between the HCW and the Other can be described in terms of Emmanuel Levinas’ face-to-face ethics where the ‘face of the Other’ only reveals itself when the Self of the HCW is disrupted and opened to receive the Other on her own terms. Levinas calls for HCWs to be responsible to the Other in a duty-conscious ethic which requires a sense of receptivity, humbleness, and willingness to learn from the Other. It also requires a dis-empowering of the HCWs own positionality. This duty-conscious ethic also underlies the concept of cultural humility [69].

#### Dialogue

The idealised form of conversation with the Other is dialogue. When this possibility opens up within the health care encounter it can be seen as an ‘ambivalent and hybrid process of meaning negotiation’ where ‘indeterminacy rather than fixity, multivocality rather than totalising unity, the production of Other ground rather than reproductive self-expression oriented to common or middle ground’ is privileged [60].

#### Operationalisation of the intercultural model

There is currently a one-day cultural awareness programme available for health personnel in Central Australia. Participants include nurses, doctors, and allied health professionals as well as non-client facing professionals such as pathologists. A recent evaluation noted that it produced short-term positive changes in participant attitudes towards cultural safety, critical thinking and transformational unlearning [70]. This one-day course cannot conceivably meet the needs of the variety of health-related professionals in their variable contexts and levels of cultural experience.

The proposed intercultural praxis model, primarily aimed at HCWs working in remote Aboriginal communities, is translatable to HCWs in regional town centres of Central Australia. It is envisaged that facilitation of the model by educators responsible for ongoing staff development will be in collaboration with local Aboriginal cultural trainers. The pedagogical disposition of these educators is critical. As Nakata argues, [71] it is inadequate for educators to fixate on a ‘simplistic decolonisation of Western knowledge and practices.’ A focus on decolonising students and their complicity with colonialism and its sequalae can tend to alienate and be counterproductive. Whilst being confronted and unsettled are appropriate reactions of people new to this material, a more productive outcome would be to facilitate critical analysis and inculcate long term reflection, a sustained conversation, with the intercultural and post/colonial Australia.

The intercultural praxis model offered here provides HCWs who have therapeutic relationships with Aboriginal people an opportunity to grapple with concepts of identity, racism and interculturality at some depth, and to focus on embedding cultural safety and humility within a critical reflexive praxis. The depth of these reflections are not required by the local pathologist whose feedback to the current programme was that ‘all bloods are the same’ [70].

## Conclusions

It is the thesis of this paper that racism is associated with fundamentalist understandings of identity and culture. Everyday racism in the form of racial microaggressions were found to be commonplace in the talk of HCWs, especially RANs, in two remote Aboriginal community health settings. This type of racism usually occurs unconsciously and is unlikely to come to consciousness in HCWs who do not adopt forms of reflexive practice or whose racist discourse goes unchallenged. Supporting remote HCWs to develop the attitudes, knowledge, and skills required to support the processes of reflexivity and positioning are a first step to providing a culturally safe clinical environment for Aboriginal people. A comprehensive model of interculturality based on the concepts of deCentred hybridity, the third space, threads and small culture formation on-the-go, combined with the duty-conscious face-to-face ethics of Emmanuel Levinas is proposed for use in the remote Central Australian setting. Given the foundations of the model it is expected to be generalisable to other remote Aboriginal health contexts. Reducing the impact of racism on Aboriginal people and strengthening intercultural dialogue are essential first principles in improving outcomes of chronic conditions such as diabetes.

The intercultural encounter lies within the sociocultural diversity of the everyday. In this sense ‘culture’ is not only referring to ethnic cultures but also to subcultures within and between ethnic groups, that represent marked ‘difference, unfamiliarity, and psychological distance’ [72] between people. Nurturing an intercultural stance as a way of being-in-the-world, [19] is fitting for HCWs in remote Aboriginal Central Australia, as well as to the wider multicultural society of Australia. It allows small culture formation on-the-go as an everyday process of respectfully encountering Others.

*I cannot approach the other, our relationship, by being rational or objective since what we do cannot but be intersubjective, intercultural...we co-perform, co-build, co-become. Interculturality is within and between you and me, us* [73].

## Electronic supplementary material

Below is the link to the electronic supplementary material.

Supplementary Material 1

## Data Availability

The participants in this study were assured of confidentiality and that access to the recorded interview data would only include the authors on this study. There is no data held in a public repository. The data is maintained in a password-protected drive at the Menzies School of Health Research.

## Abbreviations

Aboriginal Peoples: First Nations Peoples who inhabit mainland Australia and Tasmania
Indigenous Peoples (of Australia): Aboriginal and Torres Strait Islander Peoples
HCW: non-Aboriginal nurse or doctor
RAN: Remote Area Nurse
RMP: Remote Medical Practitioner
AHP: Aboriginal Health Practitioner
