# Supplementary material for: Racial microaggressions and interculturality in remote Central Australian Aboriginal healthcare
Source: Int J Equity Health. 2023 May 25;22:103. doi: 10.1186/s12939-023-01897-4 (PMC10211281; doi:10.1186/s12939-023-01897-4)
Supplement: Supplementary file 1 — Supplementary Material 1 [file 12939_2023_1897_MOESM1_ESM.pdf]

INTERVIEW GUIDE - HEALTH CARE WORKERS

BIOGRAPHICAL

- How long have you worked in remote Aboriginal health?
- How long have you worked in this particular community?
- What is the nature of your employment? Casual, Permanent, Locum?
- What services do you provide to this community?

ROLES, MOTIVATIONS & CLINIC STRUCTURES

- Why did you choose to work in remote Aboriginal Health care?
- What is your experience of working with AHPs or other Aboriginal people in the clinic?
- Have you received any training or guidance about working with Aboriginal people?
- Is there a Health Board or other community structure that has input into decisions about the clinic?
  - Who makes rules about the clinic? Eg: opening times
  - Is it important for Aboriginal people to have a say in clinic management?
  - Who do you think owns the clinic?
- Does this clinic / you go out to pick up people from camp and bring them to the clinic?
  - Is this important?
  - In what circumstances?
- Do you have relationships with Aboriginal people in this community outside of clinic hours?
- Overall, how do you perceive your role?
- Do you think local Aboriginal people see your role as you do?

COMMUNICATION

- What is the language spoken in this community?
- Have you learned any Aboriginal language?
- Do you think it is helpful to know some language?
- Do you ever use interpreters to help understand your patients?
- Do you think Aboriginal people should be made to speak English?
- Do you think non-Aboriginal people should speak an Aboriginal language?
- Have you had any training about how to speak with Aboriginal people?
- What strategies (verbal or non-verbal) do you use to communicate with Aboriginal patients?
- What style of communication works best for you?

CULTURAL & HISTORICAL KNOWLEDGE

- What orientation did you receive to work in this community?
  - Remote Aboriginal Health
  - Cultural Orientation
  - In your particular community

- Do you know anything about the history of this particular community?
- Do you think it is important to know about the history of a community/tribal group?
- How did/do you find out about the history and culture of this community?
- Do you think culture helps or hinders health in this community?
    - Are there any particular beliefs or behaviours that you have identified?
- What do you understand about cultural safety/competency?
- How do you try to ensure your practice is culturally safe?
- How does this clinic/health service ensure cultural safety?
- What do you think is your responsibility toward Aboriginal visitors from other communities?

MODELS OF HEALTH

- In general, how would you rate the health of Aboriginal people in this community?
- How do you measure health?
- What do you think causes ill health in this community?
- Do you think Aboriginal people care about their health?
- How would you rate the health literacy in this community?
- Do Aboriginal people have a different conception or model of health?
    - If so, what do you know about that?
    - If so, how do you reconcile/work within the different models?

ATTITUDES AND BEHAVIOURS TOWARD PEOPLE LIVING CHRONIC DISEASE

- Who is primarily responsible for CD/DM management?
- What are your thoughts on clinic involvement in chronic diseases such as DM?
- What attitudes and behaviours of clinical staff enable better chronic disease management?
- Have you had much success with the management of diabetes?
- What do you think is the most important part of managing people living with diabetes (PLWD)?

- What are your thoughts on the role of medication in PLWD?
- Are Aboriginal people adherent to medication?
- What strategies might/do improve adherence?
- How do you deal with people who are non-adherent?

PERSONAL WELLBEING

- How do you look after yourself working in this community?
- Have you ever experienced burnout?
- What things help to sustain you in this type of work?

If you could influence one thing to promote better health for Aboriginal people in this community what would that be?

Any other comments or issues you would like to discuss?
